# Over-the-scope clip as a rescue treatment for massive bleeding due to Dieulafoy lesion at the colorectal anastomosis: A case report

**DOI:** 10.1097/MD.0000000000037871

**Published:** 2024-04-19

**Authors:** Ping Han, Demin Li, Qiaozhen Guo, Yu Lei, Jingmei Liu, Dean Tian, Wei Yan

**Affiliations:** a Department of Gastroenterology, Tongji Hospital, Tongji Medical College, Huazhong University of Science and Technology, Wuhan, People’s Republic of China.

**Keywords:** anastomosis, case report, Dieulafoy lesion, gastrointestinal hemorrhage, over-the-scope clip

## Abstract

**Rationale:**

The bleeding of Dieulafoy lesion predominantly involves the proximal stomach and leads to severe gastrointestinal bleeding. However, these lesions have also been reported in the whole gastrointestinal tract. Bleeding of Dieulafoy lesions at the anastomosis was seldomly reported and was very easy to be ignored clinically.

**Patient concerns:**

We describe a 72-year-old woman with a past history of surgery for rectal carcinoma hospitalized with chief complaint of massive rectal bleeding. No gross bleeding lesion was found during the first emergency colonoscopy. Despite multiple blood transfusions, her hemoglobin rapidly dropped to 5.8 g/dL.

**Diagnosis:**

She was diagnosed with Dieulafoy lesion at the colorectal anastomosis during the second emergency colonoscopy.

**Interventions:**

Primary hemostasis was achieved by endoscopic hemostatic clipping. However, she experienced another large volume hematochezia 3 days later, and then received another endoscopic hemostatic clipping. She was improved and discharged. However, this patient underwent hematochezia again 1 month later. Bleeding was arrested successfully after the over-the-scope clip (OTSC) was placed during the fourth emergency colonoscopy.

**Outcomes:**

This patient underwent 4 endoscopic examinations and treatments during 2 hospitalizations. The lesion was overlooked during the first emergency colonoscopy. The second and third endoscopes revealed Dieulafoy lesion at the colorectal anastomosis and performed endoscopic hemostatic clippings, but delayed rebleeding occurred. The bleeding was stopped after the fourth emergency colonoscopy using OTSC. There was no further rebleeding during hospitalization and after 2-year of follow-up.

**Lessons:**

As far as we know, there is no reported case of lower gastrointestinal bleeding caused by Dieulafoy lesion at the colorectal anastomosis, OTSC is a safe and effective rescue treatment for Dieulafoy lesions.

## 1. Introduction

Dieulafoy lesion is a rare but well-recognized cause of severe gastrointestinal bleeding, which refers to abnormally large and tortuous primary arterial branches extending in the submucosa without caliber loss and protruding through small mucosal defects.^[1]^ Dieulafoy lesions account for approximately 5% of all causes of acute gastrointestinal bleeding, with over 70% of lesions occurring in the proximal part of the stomach.^[2]^ However, it has also been reported that these lesions occur in other parts of the gastrointestinal tract.^[3]^ Bleeding of Dieulafoy lesions at the anastomosis was only reported in a few case reports, including hepaticojejunal anastomosis,^[4]^ ileal anastomosis^[5]^ and gastrointestinal anastomosis.^[6]^ The case of Dieulafoy lesion at the gastrointestinal anastomosis reported by Gurzu et al was eventually confirmed by postoperative pathology,^[6]^ it indicates that the Dieulafoy lesion at the anastomosis is very easy to be ignored clinically.

Recently, endoscopic hemostasis has revolutionized the management to be the first-line treatment for bleeding from Dieulafoy lesions.^[7]^ However, the rebleeding rates and complete hemostasis of complicated lesions remain a challenge and can be difficult to achieve. Conventional clipping devices often appear technically insufficient to provide adequate tissue compression to obliterate the bleeding artery or rebleeding due to premature clip dropped off. The over-the-scope clip (OTSC) has brought significant improvements to these difficult situations. As compared to conventional clips, OTSC offers strong tissue apposition with greater jaw width and strength. It has unique advantages in situations of refractory gastrointestinal hemorrhage.^[8]^ However, the application of OTSC in Dieulafoy lesions has rarely been reported. In this article, we describe a case of repeated hemorrhage due to Dieulafoy lesion at the colorectal anastomosis which was failed to control by multiple endoscopic treatments and eventually rescued by endoscopic OTSC.

## 2. Case report

### 2.1. Consent

This study was approved by the Ethics Committee of Tongji Hospital, Tongji Medical College, Huazhong University of Science and Technology. Informed written consent was obtained from the patient and her relatives for publication of this case report and accompanying images.

### 2.2. Case presentation

**Figure 1.**
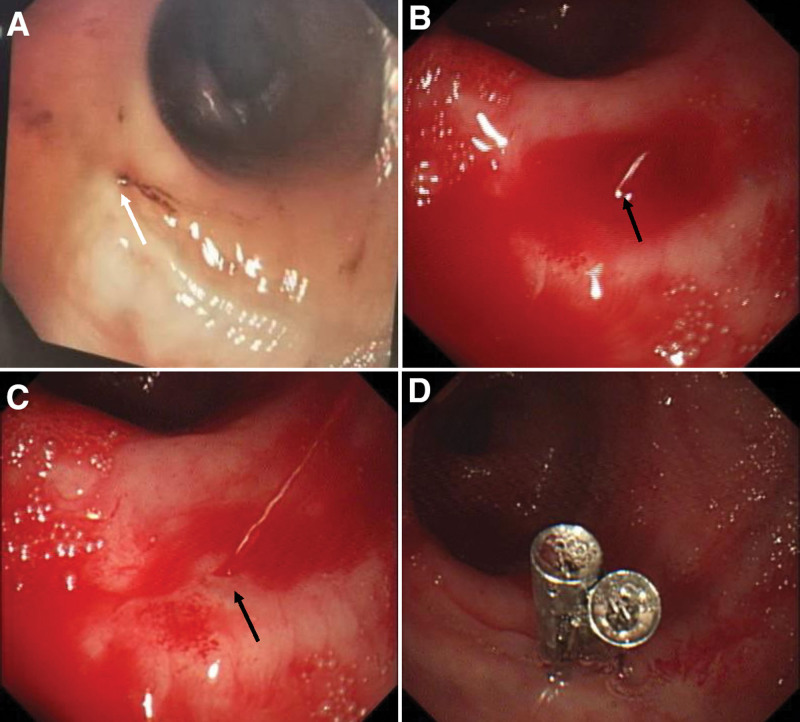
The first emergency colonoscopy (A) shows a suspicious small lesion with a little blood clot on its surface at the colorectal anastomosis (white arrow). The second emergency colonoscopy (A and B) show a single, spurt bleeding protruding from the colorectal anastomosis without an ulceration (black arrow). Immediate hemostasis achieved by endoscopic therapy with 2 hemostatic clips (C).

**Figure 2.**
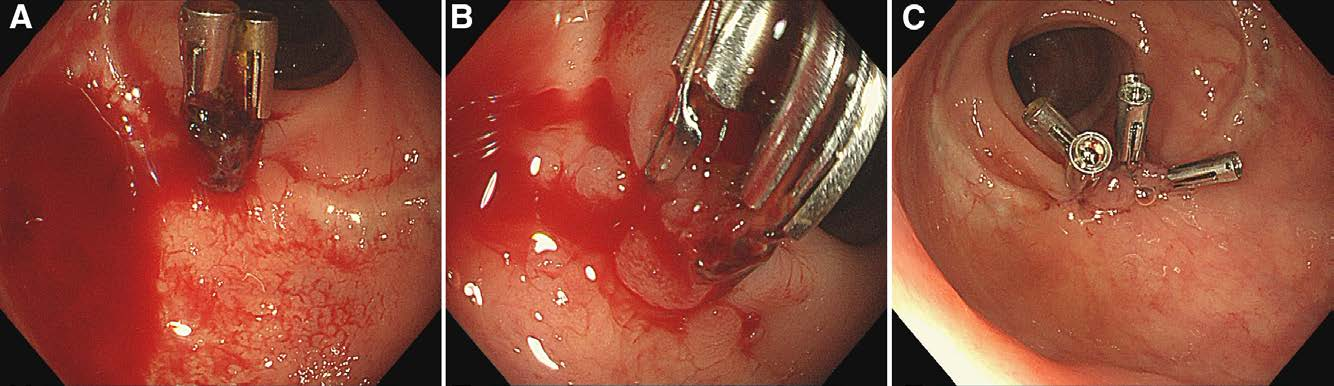
The third emergency colonoscopy. A and B suggest active arterial oozing at the Dieulafoy lesion of the colorectal anastomosis, and one clip dropped off. Another 2 hemostatic clips were performed (C).

**Figure 3.**
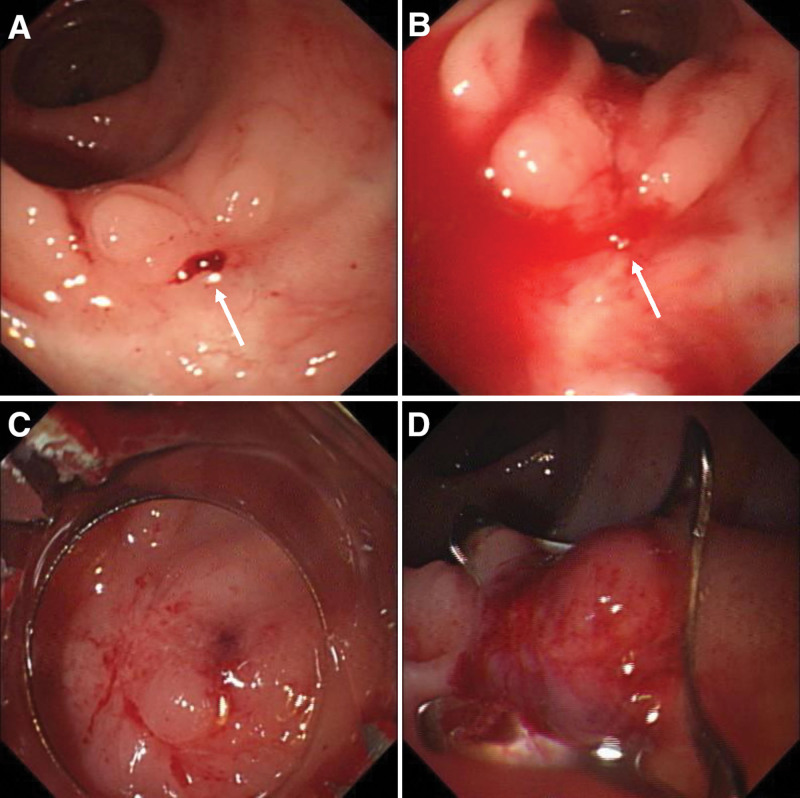
The fourth emergency colonoscopy. An adherent clot (white arrow) attached to the Dieulafoy lesion and no clip remained (A). It turned to be pulsatile bleeding (white arrow) after washed out the adherent clot (B). Successful hemostasis achieved by endoscopic OTSC (C). OTSC = over-the-scope clip.

**Figure 4.**
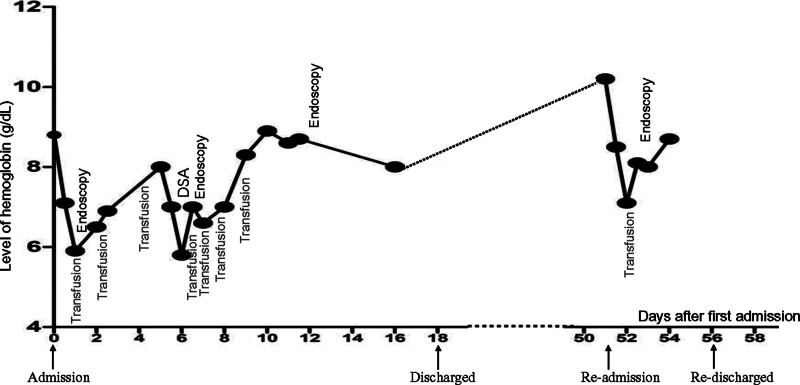
A chart for the timeline evolution of this case.

A 72-year-old female was transferred to our hospital presented with more than 10 episodes of bright red blood per rectum for 1 day. She denied nausea, fever, vomiting, or abdominal pain. She had a past history of surgery for rectal carcinoma 10 years ago. There was no history of nonsteroidal antiinflammatory drug, antiplatelet or anticoagulant drugs use. She has no history of diabetes mellitus, hypertension or chronic kidney disease. She was pale, with a pulse rate of 110 beats per minute and blood pressure of 112/86 mm Hg. Her abdomen was soft and nontender on physical examination. Laboratory test results on admission revealed hemoglobin of 8.8 g/dL. Other laboratory test results, including plasma urea nitrogen, creatinine, transaminase, platelet count, and coagulation function, were all within normal ranges. The Carbohydrate antigen 72-4(CA72-4) was slight increase at 8.01 U/mL (normal range < 6.9 U/mL), the levels of CA19-9, CA15-3, CA125, CEA and AFP are all in normal range. Abdominal computed tomography (CT) scans revealed thickening of the rectum wall. Intravenous fluid resuscitation and blood transfusion were given after admission, but she still suffered intermittent hematochezia, and her blood pressure dropped to 88/43mmHg. An emergent colonoscopic examination revealed a large amount of bright red blood and dark red blood clots in the left part of colon. Interestingly, yellow feces were appeared in the right part of colon. The anastomosis was visible at 5 cm above the anal verge, but no obvious bleeding focus was identified after careful washing. Her condition was temporarily stable after colonoscopy, but it reappeared 3 days later. Mesenteric arteriography was performed due to uncontrollable bleeding, but no active bleeding lesions were found. Despite multiple blood transfusions, her hemoglobin rapidly dropped to 5.8 g/dL. We reobserved the pictures of the first endoscopic examination, and found a suspicious small lesion with a little blood clot on its surface at the colorectal anastomosis (Fig. 1A). We carried the second emergency colonoscopy which did demonstrate a single, spurt bleeding protruding from the colorectal anastomosis without a mucosal defect or ulceration (Fig. 1B and C), consisting with a Dieulafoy lesion. Primary hemostasis was achieved after endoscopic therapy with 2 hemostatic clips (Fig. 1D). The patient did well for 3 days, and then she experienced another episode of hematochezia. The third emergency colonoscopy found active arterial oozing at the Dieulafoy lesion of the colorectal anastomosis (Fig. 2A and B). She was improved and discharged after another 2 hemostatic clips performed (Fig. 2C). However, 1 month later, she underwent hematochezia again. The fourth emergency colonoscopy found an adherent clot attachment to the Dieulafoy lesion, with no clip remained (Fig. 3A), it turned to pulsatile bleeding after washed out (Fig. 3B). Considering that this is a refractory Dieulafoy lesion, an OTSC was carried out and excellent hemostasis was obtained immediately (Fig. 3C and D). A chart presenting the timeline evolution of this case can be found in Figure 4. There was no further rebleeding during hospitalization and after 2-year of follow-up.

## 3. Discussion

Dieulafoy lesion was initially named as exulceratio simplex by Gallard in 1884, and then it has also been described as gastric aneurysm, submucosal arterial malformation, Dieulafoy ulcer, cirsoid aneurysm, and caliber persistent artery in the last 2 centuries.^[9]^ Dieulafoy lesions are majorly detected in the proximal part of stomach, particularly at the lesser curvature of the stomach within 6 cm of the gastro-esophageal junction.^[2]^ However, cases have also been documented in the whole gastrointestinal tract and outside the gastrointestinal tract, including esophagus, duodenum, jejunum, colon, rectum and bronchus.^[10]^ Dieulafoy lesions at the anastomosis were also reported in a few case reports. Cohen et al reported a 64-year-old man presented with melena and acute anemia with a history of reconstruction with esophagojejunal anastomosis and hepaticojejunal anastomosis for the surgery for pancreatic cancer, the endoscopy showed 2 angioectasias and a Dieulafoy lesion with red stigmata.^[4]^ Iwamoto et al described a 15-year-old girl with a surgical history of meconium obstruction during infancy, she rapidly developed massive bleeding due to Dieulafoy lesion from an ileal perianastomotic confirmed by surgery.^[5]^ Gurzu et al reported a 75–year–old man presented with recurrent hematemesis for 8 months with a past history of a Billroth-II gastric reconstruction for a peptic ulcer. The endoscopy suggested a type 0-IIc early gastric stump cancer at the anastomotic area, which was finally diagnosed as early gastric stump carcinoma with Dieulafoy lesion by histological examination of the surgical specimen.^[6]^ In this article, we reported a case of massive bleeding due to Dieulafoy lesion at the colorectal anastomosis, which was not discovered in the first emergency colonoscopy as the small rupture of the lesion. As a result, the Dieulafoy lesions at the anastomosis may not be apparent, more attention should be paid during an emergency colonoscopy.

The Dieulafoy lesion has a predilection for males in their fifth and sixth decades of life. In a recent review by Inayat et al, rectal Dieulafoy lesion frequently involved patients in their seventh and eighth decades of life, with an overall male-to-female ratio of 1.2:1.^[7]^ The incidence and etiology for Dieulafoy lesions are still uncertain, congenital and acquired vascular malformations are also been considered possible etiological factors for Dieulafoy lesions.^[10]^ Co-morbidities are present in 90% of patients, during which the most common comorbid conditions are hypertension, chronic renal disease, diabetes mellitus, cardiovascular and cerebrovascular diseases, and cancer. Interestingly, it has been observed that a large proportion of Dieulafoy bleeding patients are hospitalized.^[7]^ This suggests that this lesion may be attributed to stress injury and predilection for the infirm and critically ill individuals.^[10]^ Approximately half of the patients with Dieulafoy lesions received nonsteroidal antiinflammatory drugs, aspirin, or warfarin.^[11]^ This indicates that drug-induced mucosal damage may be one of the causes for the rupture and bleeding of Dieulafoy lesions.^[12,13]^ For the rectum Dieulafoy lesions, constipation and senile degenerative changes in the vascular and submucosal beds have been recognized as specific mechanical precipitating factors for the rupture of rectal Dieulafoy lesions.^[7]^ The patient in our report has no special co-morbidity except a history of surgery for rectal carcinoma 10 years ago. There was doubt about the origin of the lesion, it could have been related to neoformation of blood vessels or varicose veins as a result of the surgery, consistent with the assumption of Cohen et al.^[4]^

Endoscopy is the gold standard and the initial modality of choice for its widespread availability and easy application. The endoscopic criteria have been formulated for diagnosis of Dieulafoy lesions^[14]^: active arterial spurting or micropulsatile streaming from a minimal mucosal defect (<3 mm) or through normal surrounding mucosa; visual evidence of raised nipplelike artery within a minute mucosal defect or through normal surrounding mucosa; and blood clots with a narrow point of attachment to a minute mucosal defect or mucosa of normal appearance. The first emergency colonoscopy for our patient found no gross bleeding lesion as its relatively unobtrusive nature and feature of intermittent hemorrhage. The second emergency colonoscopy observed an active spurt bleeding at the colorectal anastomosis. The third emergency colonoscopy found an active arterial oozing, and the fourth emergency colonoscopy suggested an appearance of an adherent clot with a narrow point of attachment to a minimal mucosal defect at the colorectal anastomosis. These fit with the endoscopic features of Dieulafoy lesion.

Surgery has been the traditional treatment of this lesion, but was associated with a mortality rate up to nearly 80%.^[15]^ The therapeutic endoscopy has evolved into a feasible, safe and effective modality in recent years, with a hemostatic success rate above 90%.^[16]^ Thus, the European Gastrointestinal Endoscopy Society recommends endoscopic hemostasis for the treatment of bleeding Dieulafoy lesions,^[17]^ which includes thermal coagulation (electrocoagulation, heat probe coagulation, and argon plasma coagulation), injection (epinephrine injection or sclerotherapy), mechanical hemostasis (hemoclips or band ligation), or combined therapy. For rectum Dieulafoy lesions, endoscopic hemoclipping achieved a higher primary hemostasis rate in comparison to band ligation, but the rebleeding rates are clinically significant in both endoscopic therapeutic interventions. The OTSC system utilizes a contractile, superelastic nickel-titanium alloy, which provides superior tissue apposition than that of conventional endoclip. Although not recommended as a routine treatment for Dieulafoy lesion, OTSC can function as a “rescue therapy” in patients with severe bleeding in whom prior endoscopic therapies have failed.^[18]^ Soetikno et al used an OTSC in the treatment of 5 patients with severe bleeding from the transitional zone of the anorectum caused by hemorrhoid therapy, digital trauma, and a Dieulafoy lesion. Primary hemostasis was achieved in all the patients using one single OTSC, and no rebleeding was observed.^[19]^ In our case, bleeding of Dieulafoy lesion could not be controlled after twice endoscopic hemoclipping and angiography, which was eventually achieved by an OTSC. The cost of the OTSC is approximately 10 to 30 times higher than the cost of a through-the-scope clip in China, this is one of the important reasons that limit its widely application.

## 4. Limitations of the study

Angiography is recommendable for bleeding of Dieulafoy lesion, but in this case, no active bleeding lesion was detected by angiography. After the second endoscopic hemostasis, DSA embolization might be a good option, as even without active bleeding, the metal clip can serve as a localization marker for the lesion. More studies are needed to evaluate the diagnostic and therapeutic effects of DSA and endoscopy for Dieulafoy lesions at the anastomosis

## 5. Conclusion

In conclusion, we report massive bleeding due to Dieulafoy lesion at the colorectal anastomosis in an older patient, and she was finally rescued by an endoscopic OTSC without rebleeding during follow-up. The OTSC system represents an effective, easily performed, and safe endoscopic therapeutic which should be taken as a “rescue therapy” when conventional endoscopic techniques have failed.

## Author contributions

**Conceptualization:** Ping Han, Demin Li, Qiaozhen Guo, Wei Yan.

**Data curation:** Qiaozhen Guo, Yu Lei.

**Investigation:** Ping Han, Demin Li, Qiaozhen Guo, Jingmei Liu.

**Methodology:** Yu Lei, Jingmei Liu.

**Project administration:** Ping Han.

**Resources:** Demin Li.

**Visualization:** Dean Tian, Wei Yan.

**Writing – original draft:** Ping Han.

**Writing – review & editing:** Dean Tian, Wei Yan.
